# Three hematological indexes that may serve as prognostic indicators in patients with primary, high-grade, appendicular osteosarcoma

**DOI:** 10.18632/oncotarget.17811

**Published:** 2017-05-11

**Authors:** Keqi Hu, Zhan Wang, Peng Lin, Zuojun Wen, Haiyong Ren, Lingling Sun, Hengyuan Li, Binghao Li, Shengdong Wang, Xingzhi Zhou, Siyuan Tengwang, Langhai Xu, Zhaoming Ye

**Affiliations:** ^1^ Center for Orthopedic Research, Orthopedics Research Institute of Zhejiang University, Department of Orthopedics, The Second Affiliated Hospital of Zhejiang University School of Medicine, Hangzhou, 310000, China; ^2^ Department of Orthopedics, Tongde Hospital of Zhejiang Province, Hangzhou, 310000, China

**Keywords:** osteosarcoma, prognosis, alkaline phosphatase, lactate dehydrogenase, fibrinogen

## Abstract

**Background:**

Although alkaline phosphatase (ALP) and lactate dehydrogenase (LDH) are considered effective prognostic factors of osteosarcoma, useful prognostic biomarkers for patients with osteosarcoma are still lacking.

**Methods:**

A retrospective study of 106 patients with primary, high-grade, appendicular osteosarcoma obtained between 2006 and 2011 was performed to assess the prognostic value of serum ALP, LDH and fibrinogen (FBG) levels, as well as their decrease rates in osteosarcoma. The Kaplan-Meier method was employed to analyze overall survival. The Cox proportional hazard model was used to determine the significance of these prognostic biomarkers on survival distribution.

**Results:**

Patients with pre-ct (before neoadjuvant chemotherapy) LDH>210U/L, post-ct (after neoadjuvant chemotherapy, but before surgery) LDH>215U/L, post-ct FBG>2.8g/L, FBG DR (Decrease Rate)≤10% tended to have a poorer prognosis.

**Conclusions:**

High pre-ct and post-ct peripheral serum LDH level, high serum post-ct FBG level and low decrease rate of serum FBG were related to poor survival in patients with osteosarcoma. Fibrinogen was found to be a new valuable predictor of 5-year survival in patients with osteosarcoma for the first time.

## INTRODUCTION

Osteosarcoma is the most common primary malignant bone tumor, and accounts for less than 1% of cancers overall [1]. Despite improvements in the outcome over the past 30 years for patients with local disease, the 5-year survival rate for patients with metastatic disease at presentation or recurrent disease remains poor [2]. Therefore, to improve the prognosis of high-risk patients, finding valuable prognostic factors is particularly important.

Alkaline phosphatase (ALP) is considered to be clinically significant when making a diagnosis or predicting the prognosis of patients with osteosarcoma [3–6]. Lactate dehydrogenase (LDH) is a significant prognostic biomarker in several tumors, including pancreatic cancer, lung cancer, rectal cancer, prostate cancer and hematological malignancies, as well as in bone tumors such as Ewing's tumors and osteosarcomas [7]. Although ALP and LDH are considered effective prognostic factors of osteosarcoma, prognostic biomarkers for patients with osteosarcoma are still lacking. Fibrinogen (FBG) is an acute phase glycoprotein that is traditionally associated with the maintenance of hemostasis. Recently, more and more evidence suggests that plasma FBG is an important factor in both inflammation and cancer progression [8]. Moreover, clinical studies have indicated that high pre-treatment plasma FBG level is related with poorer overall survival and disease-specific survival in various types of cancers, such as pancreatic cancer [9], lung cancer [10], colon cancer [11], etc. However, the relationship between plasma FBG level and the prognostic significance in patients with osteosarcoma has not been evaluated.

Additionally, many studies have focused on the prognostic value of pre-treatment (before neoadjuvant chemotherapy) ALP or LDH in osteosarcoma. Therefore, we decide to identify the prognostic significance of serum ALP, LDH, FBG and their decrease rates in patients with osteosarcoma, both before and after neoadjuvant chemotherapy.

## RESULTS

### Clinical data

Clinical characteristics of the 106 patients in our study were shown in Table 1. The mean age was 19 years (ranging from 7 to 53 years), and the mean follow-up was 52 months (ranging from 7 to 80 months). 62 patients were male (58.5%) and 44 patients were female (41.5%). The most common location of tumor is femur (68 cases). Other locations include tibia (26 cases), humerus (12 cases) and fibula (2 cases). 20 patients (18.9%) had metastases during the follow-up time, including pulmonary metastases of (14 cases) and bone metastases (6 cases). 12 patients (11.3%) had pathological fractures during the follow-up.

**Table 1:** Clinical parameters of 106 patients with osteosarcoma

Variable	Number	Percent
Gender		
Female	44	58.5%
Male	62	41.5%
Age		
Mean age	19(7-53)	
>20 years	24	22.6%
≤20 years	82	77.4%
Metastasis		
Pulmonary metastasis	14	13.2%
Bone metastasis	6	5.7%
No metastasis	86	81.1%
Site		
Femur	68	64.2%
Tibia	26	24.5%
Humerus	12	9.4%
Fibula	2	1.9%
Pathologic fracture		
Yes	12	11.3%
No	94	88.7%

### Cut-off point

**Figure 1:**
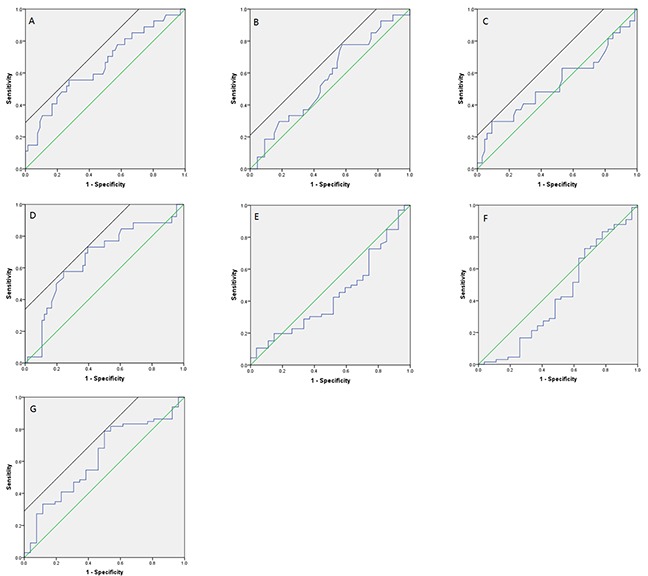
ROC curve for predicting patients’ prognosis with hematologic indexes **(A)** Pre-ct LDH; **(B)** Pre-ct FBG; **(C)** Post-ct LDH; **(D)** Post-ct FBG; **(E)** ALP DR; **(F)** LDH DR; **(G)** FBG DR.)

ROC curves of hematologic index predicting whether patients would die of osteosarcoma within 56 months were shown in Figure 1. According to those ROC curves, the critical points were determined: 210U/L for pre-ct LDH (Figure 1-A), 3.0g/L for pre-ct FBG (Figure 1-B), 215U/L for post-ct LDH (Figure 1-C), 2.8g/L for post-ct FBG (Figure 1-D), and 10% for FBG DR (Figure 1-G). Since the ROC curves of ALP DR (Figure 1-E) and LDH DR (Figure 1-F) were not good enough to choose a critical point, 50% for ALP DR, 10% for LDH DR were chose according to our clinical experience.

### Statistical outcome

The results of Chi-square test analyzing the difference of clinical parameters (gender, age at diagnosis, tumor location, metastasis, pathological fracture) between two groups to test the comparability were shown in Table 2–6. Patients with metastasis tend to have higher pre-ct ALP and LDH (Table 2). Patients ≤20 years old were more likely to have high pre-ct LDH (Table 2), whereas patients >20 years old were correlated with low ALP DR and low LDH DR (Table 5). Female patients had higher post-ct FBG (Table 4). Therefore, gender, age and metastasis interfered the comparability of two groups and then the multivariate analysis of these factors was performed later.

**Table 2:** Comparability of groups with different levels of pre-ct ALP and pre-ct LDH

Factor	Low pre-ct ALP	High pre-ct ALP	P value	Low pre-ct LDH (Mean value: 165.0U/L)	High pre-ct LDH (Mean value: 278.6U/L)	P value
				Cut-off value: 210U/L		
Gender						
Female	33	11		30	14	
Male	47	15	0.924	40	22	0.695
Age						
≤20 years	62	20		50	32	
>20 years	18	6	0.951	20	4	0.042
Metastasis						
No	70	16		62	24	
Yes	10	10	0.007	8	12	0.006
Pathologic fracture						
No	72	22		64	30	
Yes	8	4	0.483	6	6	0.331

**Table 3:** Comparability of groups with different levels of pre-ct FBG and post-ct ALP

Factor	Low pre-ct FBG (Mean value: 2.50g/L)	High pre-ct FBG (Mean value: 3.87g/L)	P value	Low post-ct ALP	High post-ct ALP	P value
	Cut-off value: 3.0g/L					
Gender						
Female	18	26		41	3	
Male	33	29	0.211	62	0	0.069
Age						
≤20 years	40	42		81	1	
>20 years	11	13	0.799	22	2	0.128
Metastasis						
No	40	46		84	2	
Yes	11	9	0.494	19	1	0.470
Pathologic fracture						
No	46	48		91	3	
Yes	5	7	0.635	12	0	1.000

**Table 4:** Comparability of groups with different levels of post-ct LDH and post-ct FBG

Factor	Low post-ct LDH (Mean value: 151.5U/L)	High post-ct LDH (Mean value: 287.0U/L)	P value	Low post-ct FBG (Mean value: 2.11g/L)	High post-ct FBG (Mean value: 3.66g/L)	P value
	Cut-off value: 215U/L			Cut-off value: 2.8g/L		
Gender						
Female	39	5		24	20	
Male	51	11	0.366	48	13	0.009
Age						
≤20 years	70	12		57	24	
>20 years	20	4	0.755	15	9	0.466
Metastasis						
No	76	10		57	28	
Yes	14	6	0.075	15	5	0.491
Pathologic fracture						
No	79	15		65	28	
Yes	11	1	0.688	7	5	0.511

**Table 5:** Comparability of groups with different levels of decrease rate of ALP and LDH

Factor	High ALP DR (Mean value: 67.84%)	Low ALP DR (Mean value: 27.39%)	P value	High LDH DR (Mean value: 33.03%)	Low LDH DR (Mean value: -19.83%)	P value
	Cut-off value: 50%			Cut-off value: 10%		
Gender						
Female	22	22		25	19	
Male	34	28	0.623	33	29	0.714
Age						
≤20 years	53	29		50	32	
>20 years	3	21	0.000	8	16	0.017
Metastasis						
No	42	44		47	39	
Yes	14	6	0.088	11	9	0.977
Pathologic fracture						
No	49	45		50	44	
Yes	7	5	0.685	8	4	0.377

**Table 6:** Comparability of groups with different levels of decrease rate of FBG

Factor	High FBG DR (Mean value: 30.07%)	Low FBG DR (Mean value: -21.46%)	P value
	Cut-off value: 10%		
Gender			
Female	29	15	
Male	47	14	0.208
Age			
≤20 years	59	22	
>20 years	17	7	0.847
Metastasis			
No	61	24	
Yes	15	5	0.771
Pathologic fracture			
No	68	25	
Yes	8	4	0.733

**Figure 2:**
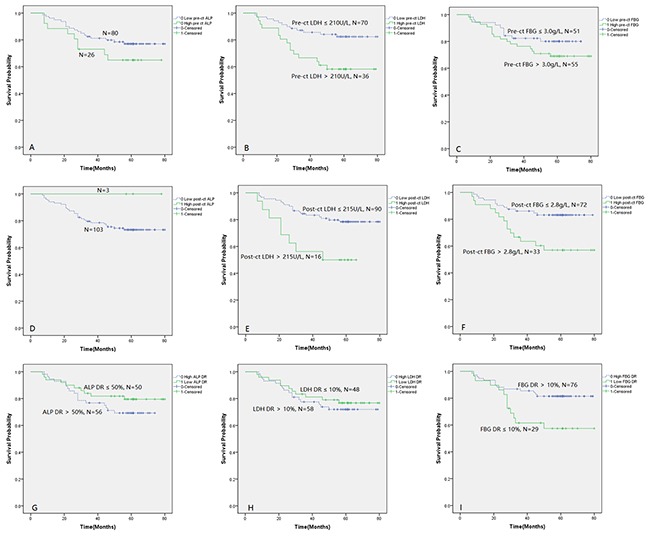
Kaplan-Meier analysis showing survival difference between groups divided by levels of hematologic indexes or their decrease rates (**A**: Pre-ct ALP; **B**: Pre-ct LDH; **C**: Pre-ct FBG; **D**: Post-ct ALP; **E**: Post-ct LDH; **F**: Post-ct FBG; **G**: ALP DR; **H**: LDH DR; **I**: FBG DR.)

The consequences of Kaplan-Meier survival analysis were shown in Figure 2. It was found that patients with pre-ct LDH>210U/L had 5-year survival rate of 58% (95% CI, 42%–74%), which was better than patients with pre-ct LDH≤210U/L having 82% (95% CI, 73%–91%) (log rank test, p = 0.006) (Figure 2-B). Patients with post-ct LDH>215U/L had 5-year survival rate of 50% (95% CI, 26%–75%), better than patients with post-ct LDH≤215U/L having 78% (95% CI, 70%–87%) (log rank test, p = 0.006) (Figure 2-E). Patients with post-ct FBG>2.8g/L had 5-year survival rate of 57% (95% CI, 40%–74%), better than patients with post-ct FBG≤2.8g/L having 83% (95% CI, 75%–92%) (log rank test, p = 0.004) (Figure 2-F). Patients with FBG DR>10% had 5-year survival rate of 57% (95% CI, 39%–76%), better than patients with FBG DR≤10% having 81% (95% CI, 73%–90%) (log rank test, p = 0.014) (Figure 2-I).

Given the results of Chi-square analyses before, gender, age and metastasis were involved as variables in Cox proportional hazard model. Since patients with metastasis and patients ≤20 years old were more likely to have high pre-ct LDH (Table 2), metastasis, age and pre-ct LDH were put together in the Cox proportional hazard model to validate their independent influence on survival (Table 7). Using the same method, gender and post-ct FBG (Table 4) were put together in the Cox proportional hazard model (Table 8). In the end, pre-ct LDH, post-ct LDH, post-ct FBG and FBG DR were put together in the Cox proportional hazard model to detect certain relationship among them (Table 9).

**Table 7:** Multivariate analysis of survival containing metastasis, age and pre-ct LDH

Factor	Hazard ratio	95% CI	P value
Metastasis	0.545	0.238-1.251	0.152
Age	3.202	0.744-13.770	0.118
Pre-ct LDH	0.463	0.210-1.025	0.057

**Table 8:** Multivariate analysis of survival containing gender and post-ct FBG

Factor	Hazard ratio	95% CI	P value
Gender	0.733	0.325-1.651	0.453
Post-ct FBG	0.315	0.141-0.704	0.005

**Table 9:** Multivariate analysis of survival containing pre-ct LDH, post-ct LDH, post-ct FBG and FBG DR

Factor	Hazard ratio	95% CI	P value
Pre-ct LDH	0.333	0.151-0.731	0.006
Post-ct LDH	0.271	0.116-0.634	0.003
Post-ct FBG	0.392	0.155-0.995	0.049
FBG DR	0.627	0.247-1.595	0.328

The multivariate analyses show that pre-ct LDH, post-ct LDH, post-ct FBG were the independent predictors of survival in osteosarcoma. While the p value of pre-ct LDH in the multivariate analyseis 0.057 (Table 7), pre-ct LDH is still considered as a significant independent predictor because of the relatively small sample of our research.

### Summary

In summary, pre-ct LDH>210U/L, post-ct LDH>215U/L, post-ct FBG>2.8g/L, and FBG DR≤10%, patients with primary, high-grade, appendicular osteos-arcoma tended to have a poorer prognosis.

## DISCUSSION

Osteosarcoma is usually of high grade malignancy. The multimodal treatment approaches of osteosarcoma from the late 1970s led to the increased overall survival [12]. However, there was no improvement in the overall survival since the 1990s among all age groups [13]. A recent review concluded that modern chemotherapy in conjunction with surgery had reached a plateau phase since the 1980s, with about 60–70% five-year event free survival in extremity localized non-metastatic disease, while other groups suffered poorer prognosis [14]. Metastasis has been proved to be a significant prognostic factor of tumors. Osteosarcoma patients with metastatic disease have overall survival of roughly 20% to 30% compared with 70% to 80% in non-metastatic patients [15]. Chemotherapy response (tumor necrosis percent) also plays an important role in prognosis of osteosarcoma. The overall and disease-specific survival of patients with larger than 90% necrosis were much improved compared to those with poorer histologic response to chemotherapy [16]. Moreover, the size and location are valuable prognostic factors for osteosarcoma. Axial tumors tend to be larger at diagnosis, and thus are associated with poorer overall survival than tumors in the extremity [14,16,17].

Although many factors can show good prognostic significance for osteosarcoma, they are not usually practically useful. Obvious metastasis can be detected through various imaging, but metastatic lesions that are too tiny to be detected can be automatically ignored. Although, chemotherapy response is valuable, it takes much efforts and time and is not routinely examined in all hospitals including our hospital. Hence, information in predicting the prognosis of osteosarcoma patients is actually lacking, which possibly partly accounts for the plateau phase of survival from osteosarcoma during last two decades. That is why ALP and LDH came in sight [3–7]. Inspired by those studies, our current research mainly focuses on using hematologic indexesto predict the prognosis of osteosarcoma. These indicators are routinely inspected after patients being admitted to hospital and the results are easy to obtain. The tests are simple, universally available, economically frugal and their results can be dynamically monitored for better prognosis of outcome.

Osteosarcoma is characterized by production of osteoid tissue or immature bone tissue [12]. Biochemical markers which reflect bone turnover activity are sensitive indicators of early bone diseases. Among them, ALP, produced by the osteoblastic cells is considered clinically useful [6]. A lot of studies have confirmed its prognostic significance in osteosarcoma [3–6]. As serum ALP is produced by various tissues, confounding diseases such as liver and kidney dysfunction in patients have to be ruled out. Nevertheless, it is quite a pity that in our study no correlation was found between ALP and osteosarcoma patients. The reason may lie in the limited number of patients involved. The critical point of abnormal ALP in our study is stricter, when Han Ju et al [5] set 150 U/L as the upper normal serum ALP limit in patients less than 18 years, and 110 U/L in those 18 years or older, which might also influence the analysis and led to no significance.

Serum LDH is involved in the interconversion of lactate and pyruvate, providing NAD+ for continued glycolysis in active muscles and is needed for long-chain fatty acid oxidation in liver peroxisomes [7]. Different from normal cells, cancer cells prefer to metabolize glucose by glycolysis to generate sufficient energy for the demands of rapid proliferation, even in the presence of adequate oxygen, which is known as the Warburg effect [14], so LDH is produced substantially by tumor and it has become a widely acceptable index that reflects tumor burden. It has been used to predict the prognosis of patients with various malignancies such as colorectal cancer [15], pancreatic cancer [16], urothelial carcinoma [17], as well as osteosarcoma [7]. Bacci G et al [18] and Chen Jian [7] have validated the prognostic value of pre-treatment serum LDH. However, little attention has been payed to the dynamics of LDH. In our study, LDH was a significant prognostic factor in patients with osteosarcoma, not only before neoadjuvant chemotherapy (pre-ct), but also after neoadjuvant chemotherapy, before surgery (post-ct). However, the decrease rate (DR) failed to show any significance of prognosis in the present study.

FBG was also demonstrated useful in prognosis of osteosarcoma in our research for the first time. However, the mechanism of increased fibrinogen in tumor has not been clarified. Sun et al. showed that highly concentrated FBG increased induced Epithelial-Mesenchymal Transitions (EMT), which plays a central role in tumor progression, including migration, invasion, metastatic capacity and multidrug resistance [19]. Fibrinogen releases proliferative signals to supplying as a scaffold for binding growth factors, such as VEGF, which accelerate cellular adhesion, proliferation and migration during angiogenesis and cancer cell growth [20]. Sahni A et al. demonstrated the possibility that tumor cells themselves may generate fibrinogen [21]. It is now generally accepted that interaction exists amongst coagulation, inflammation and cancer development. In tumor microenvironment, FBG plays an important role in inflammatory response and tumor stroma formation that are tightly correlated with cancer development and progression [8].

Our results revealed distinct effect of LDH and FBG on survival of osteosarcoma patients, which may help assign patients into different categories. Due to their substantial prognostic value, serum LDH and plasma FBG could be integrated in existing prognosis evaluating systems to further improve the precision of prognosis prediction, treatment decision-making, and risk-adjusted follow-up in patients with osteosarcoma. With the prognosis information before surgery, subsequent treatment strategy for those patients with poorer survival can be adjusted, or they can be introduced into clinical trials for novel treatments to improve their outcomes.

There were several limitations to our study. First, it was a retrospective analysis in which we could not control any confounding factors. Second, we had limited numbers of patients since osteosarcoma is a rare disease. Third, some patients were lost in follow-up and some patients’ information failed to be obtained, which added inaccuracy to this research. Fourth, in our study, we just validated LDH and FBG as a robust prognostic biomarker in osteosarcoma. They cannot be used to draw conclusions on how treatment should be adjusted for an individual patient.

In summary, the current study demonstrates the dynamic level of LDH and FBG with their decrease rates are significant prognostic factors for the survival of osteosarcoma patients. However, the specific mechanism of their role in survival still needs more research. In the future, prospective research with a larger number of patients can also be conducted to further evaluate their roles.

## PATIENTS AND METHODS

### Patients’ information

Our study included 106 patients with primary, high-grade osteosarcoma, treated at the Second Affiliated Hospital, Zhejiang University School of Medicine from December 2006 to December 2011. The inclusion criteria are: (1) The patients were diagnosed definitely through pathologic specimen and the tumor site is in extremity; (2) The patients didn't receive any treatment such as surgery, chemotherapy and radiotherapy prior to admission for osteosarcoma treatment at the first time; (3) All the patients admitted received standard regular neoadjuvant chemotherapy (MAP regimen) and sequential treatment including resection of the tumor, and post-operative chemotherapy. While the exclusion criteria are: (1) Patients were suffering liver or kidney disease, coagulation disorder, infection, hematologic system or other systemic diseases that might affect the hematologic indexes involved. (2) The relevant data and information of the treatment of the patients were unavailable. (3) Patients died of unrelated causes. This retrospective research was approved by and performed in accordance with our institutional review board.

All patients’ information including age, gender, tumor features, metastases, histologic subtype, laboratory tests, and specific treatment strategy, both before neoadjuvant chemotherapy (pre-ct) and after neoadjuvant chemotherapy, but before surgery (post-ct) were recorded carefully. If patients died during the follow-up period, survival time was confirmed from the time of first diagnosis of osteosarcoma to the death time. If patients were lost during the follow-up, “censored” was recorded with the last follow-up time. Patients were also censored at the last follow-up if no event occurred. The serum ALP, LDH and FBG levels were routinely tested both before and after neoadjuvant chemotherapy.

### Setting cut-off point

In our research, Decrease Rate (DR) of serum ALP, LDH and FBG levels was calculated through the formula: DR=[(pre-ct value)-(post-ct value)]/(pre-ct value). Serum ALP, LDH, FBG levels and their decrease rate were used to predict whether patients would die of osteosarcoma within 56 months and then the Receiver Operating Characteristic curve (ROC curve) was drawn. According to the curve, cut-off value is determined when Youden's Index (Sensitivity+Specificity-1) is the biggest. Therefore, we obtained the best critical value that may show the biggest prognostic significance.

However, ALP level is different from other indexes, since in normal population, it varies substantially with age. There is a rise at the beginning of the childhood and teenage, whereas in the middle of the childhood and adult period, the ALP level decreases. In our study, to set the critical point, we chose the abnormal ALP level standard put forward by Bacci, et al [22], slightly amended by our laboratory (2-10 years old, > 350 IU/L; female aged 10 to 13, > 400 IU/L; male aged 13 to 15, > 500 IU/L; the rest of adolescent population, > 300 IU/L; more than 20 years old, > 140 IU/L).

### Statistical analysis

With the critical values above, all the patients were divided into two groups. Chi-square test was performed to analyse the difference of clinical parameters (gender, age at diagnosis, tumor location, metastasis, pathological fracture) between two groups in order to test the comparability.

Survival is determined by Kaplan-Meier survival analysis and compared between groups with a log rank test. The Cox proportional hazard model was used to further determine the effect of the three biomarkers on survival distribution using multivariate analyses. The hazard ratios (HRs) with their 95% CIs and the p values were determined. Data analyses were performed using the SPSS 20.0 statistical software package (SPSS Inc, Chicago, IL, USA) and Microsoft Excel 2016. A significance level of *P* < 0.05 was assumed to denote difference.

### Consent

This study was approved by the local ethical (Review Board of the Second Affiliated Hospital of Zhejiang University School of Medicine).

## CONCLUSION

High pre-ct and post-ct peripheral serum LDH level, high serum post-ct FBG level and low decrease rate of serum FBG were related to poor survival in patients with osteosarcoma. Fibrinogen may also serve as a new valuable predictor of 5-year survival in patients with osteosarcoma.
